# 

**Published:** 1899-11

**Authors:** 


PUBLISHED MONTHLY.

### ATLANTA JOURNAL-RECORD OF MEDICINE.

Successor to Atlanta Medical and Surgical Journal, Established 1855, and Southern Medical Record, Established 1870.

Vol. I. Atlanta, Ga., November, 1899. No. 8.

